# Lactate Metabolism‐Immune Regulation‐Related Gene Signature in Lower‐Grade Gliomas: Prognostic Model Development and Immune Characterization

**DOI:** 10.1096/fj.202503738RR

**Published:** 2026-03-18

**Authors:** Enhao Zhang, Liangzhe Wei, He Ren, Jianfei Zhang, Hongqiao Yang, Meng Sun, Xiang Gao, Yi Huang

**Affiliations:** ^1^ Ningbo Key Laboratory of Nervous System and Brain Function, Department of Neurosurgery The First Affiliated Hospital of Ningbo University Ningbo Zhejiang China

**Keywords:** immune microenvironment, lactate metabolism‐immune regulation‐related genes, low‐grade glioma, *PLA2G2A*, prognostic model, tumor mutation burden

## Abstract

Low‐grade gliomas represent a diverse category of central nervous system tumors, characterized by considerable variability in prognosis. Metabolic reprogramming, particularly lactate metabolism, has been associated with the tumor immune microenvironment; however, the prognostic significance of the related genes in LGG is still not well understood. This study used TCGA and CGGA cohorts to perform consensus clustering based on genes involved in lactate metabolism. A predictive model was constructed using differential analysis, Cox regression, and LASSO methods. The model's association with immune cell infiltration, TMB, and drug responsiveness was also evaluated. The function of the key gene *PLA2G2A* was validated using RT‐qPCR, Western blot, immunofluorescence, ELISA, CCK‐8, Transwell assays, and mouse tumorigenesis experiments. Consensus clustering classified LGG into two distinct subtypes, each showing significant variations in survival and immune profiles. The three‐gene signature (*VAV3*, *TNFRSF12A*, *PLA2G2A*) effectively distinguished high‐risk from low‐risk patients, demonstrating high predictive accuracy in both TCGA and CGGA datasets. Patients categorized as high‐risk showed reduced immune infiltration, increased TMB, and a worse prognosis. Additionally, they showed increased sensitivity to tyrosine kinase inhibitors and platinum‐based therapies. Functional experiments showed that *PLA2G2A* was highly expressed in gliomas, and its downregulation notably reduced tumor cell proliferation, migration, invasion, and growth in vivo, while reversing the epithelial‐mesenchymal transition. This study proposes and validates a novel three‐gene prognostic model that reflects the metabolic‐immune interactions in LGG, predicts patient prognosis, and suggests potential therapeutic targets. The functional validation of *PLA2G2A* further underscores its potential as a therapeutic biomarker.

## Graphical abstract

Lactate metabolism‐immune regulation‐related gene signature (*PLA2G2A*, *TNFRSF12A*, *VAV3*) predicts glioma prognosis. *PLA2G2A* promotes ECM remodeling, tumor‐associated inflammation, and EMT, leading to enhanced proliferation, invasion, and unfavorable outcomes.

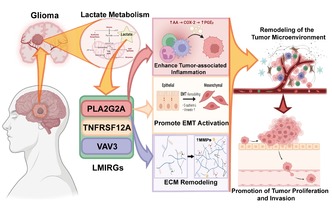

## 1 Introduction

Low‐grade glioma (LGG) is a frequently occurring, diffuse tumor within the central nervous system, encompassing WHO grades II‐III astrocytomas, oligodendrogliomas, and oligoastrocytomas [1]. LGG typically occurs in young adults and often presents with seizures as the first symptom. Although it grows more slowly than high‐grade gliomas, it is prone to recurrence and eventually progresses to glioblastoma (GBM), leading to a poor prognosis [2, 3]. Current treatment mainly involves surgical resection, supplemented by radiotherapy and chemotherapy (e.g., temozolomide). However, due to its heterogeneity and recurrence, the therapeutic efficacy remains limited [4]. Determining novel molecular biomarkers and predictive models for tailored therapy is a crucial challenge and area of emphasis in medical research.

Tumor metabolic reprogramming is a fundamental hallmark of cancer [5], with lactate metabolism playing a key role in tumor progression [6]. Even in the presence of oxygen, tumor cells tend to rely on the glycolytic pathway (the Warburg effect), resulting in a build‐up of lactate [7]. An overabundance of lactate can facilitate immune evasion and tumor invasion by acidifying the microenvironment, promoting angiogenesis, and impairing immune effector cell function [8]. In gliomas, lactate metabolism is closely associated with an immunosuppressive microenvironment, and its downstream effects may significantly impact patient survival outcomes.

Recent studies have also highlighted the emerging role of lactylation, a post‐translational modification mediated by lactate, which further connects lactate metabolism to tumor biology. Lactylation has been shown to regulate gene expression and cellular functions, including immune response modulation and metabolic reprogramming in tumor cells. Zhang et al. demonstrated that lactylation could influence the immune landscape and contribute to tumor progression [9], suggesting its potential as an additional therapeutic target in cancers, including gliomas.

The tumor microenvironment (TME) is crucial in influencing the biological characteristics of LGG [10, 11]. The tumor microenvironment (TME) comprises immune, stromal, and endothelial cells alongside the extracellular matrix, collectively impacting tumor growth, invasion, and treatment response [12, 13, 14, 15]. Research has indicated that immune infiltration in LGG is typically minimal, marked by a lack of CD8+ T cells and heightened activity of Tregs and M2 macrophages, which contributes to an immunosuppressive environment. Lactate accumulation further impairs immune cell function, contributing to the formation of the “cold tumor” phenotype [16]. Therefore, exploring how Lactate Metabolism‐Immune Regulation‐Related Genes (LMIRGs) interact with the immune microenvironment is essential for revealing the molecular mechanisms of LGG and improving prognostic assessments.

This study extensively examined the expression patterns and clinical significance of LMIRGs within LGG. We examined their stratification potential via consensus clustering, developed and validated an LMIRGs risk score model, and further investigated its association with immune cell infiltration, tumor mutational burden (TMB), and drug response. Moreover, we confirmed the functional role of key genes in the model through both in vitro and in vivo studies, aiming to provide new molecular insights for personalized treatment and prognosis in LGG.

## 2 Materials and Methods

### 2.1 Data Collection and Preprocessing

Gene expression data (fragments per kilobase of transcript per million mapped reads, FPKM), somatic mutations, and clinical information for LGG were sourced from The Cancer Genome Atlas (TCGA) via the GDC database (https://portal.gdc.cancer.gov/) [17]. Samples from the Chinese Glioma Genome Atlas (CGGA_693, CGGA_325) served as the validation cohort after batch correction [18]. The study incorporated patients with accessible survival information, yielding a final count of 510 individuals from the TCGA‐LGG dataset for the training group, alongside 592 patients sourced from the CGGA_693 and CGGA_325 cohorts for validation (Table 1). One hundred eighty‐five genes associated with lactate were identified from previous studies [19], and their details can be found in Table S1.

**TABLE 1:** Clinical data of patients included in the bioinformatics database.

Clinicopathological features		Training cohort	Validation cohort
		TCGA (*n* = 510)	CGGA (*n* = 592)
Age (years)	≤ 40	239	307
	> 40	270	284
	NA	1	1
Gender	Male	283	341
	Female	227	251
Grade	II	246	270
	III	264	322
IDH status	Mutant	89	415
	Wild‐type	33	138
	NA	388	39
1p19q codeletion status	Non‐codeletion	2	372
	Codeletion	1	180
	NA	507	40
MGMT promoter satuts	Methylated	NA	285
	Un‐methylated	NA	200
	NA	NA	107
PR type	Primary	NA	408
	Recurrent	NA	184

### 2.2 Consensus Clustering

Gene expression related to lactate metabolism was analyzed via consensus clustering with the ConsensusClusterPlus R software package. Pearson correlation was employed as the distance metric, and the clustering method used was PAM 1000 resampling cycles were conducted to verify classification consistency. The ideal cluster count was determined through the consensus matrix, the cumulative distribution function (CDF), and monitoring the variation in the CDF's area. The “survival” R package was applied to evaluate the relationship between clustering and total survival. Pheatmap, survival, and survminer R packages were employed to create heatmaps and Kaplan–Meier survival curves. The CIBERSORT algorithm functioned like a precision tool, meticulously analyzing the varying proportions of immune cell subtypes within the given sample groups. This approach revealed intricate shifts in immune cell infiltration dynamics, offering valuable insights into the underlying biological mechanisms [20].

### 2.3 Differential Gene Screening and Enrichment Analysis

Gene expression differences were assessed with the “limma,” “edgeR,” “DESeq2,” and “Wilcoxon” packages. Genes with a log‐fold change exceeding 2 and a false discovery rate under 0.05 categorize as differentially expressed across the two clusters. The genes that were expressed differently were then put through a process called Gene Ontology (GO) and Kyoto Encyclopedia of Genes and Genomes (KEGG) enrichment analysis, using the “Clusterfiler” R program. To delve into the signaling pathways, we employed Gene Set Enrichment Analysis (GSEA) [21].

### 2.4 Development and Validation of the LMIRGs Signature

Prognostically relevant DEGs were identified through univariate Cox analysis, setting a significance cutoff at *p* < 0.05. Subsequently, we employed LASSO regression followed by multivariate Cox analysis to pinpoint the most relevant genes. This rigorous screening process yielded three key biomarkers, which together form a distinct molecular signature known as LMIRGs. The risk score was calculated by multiplying each gene's coefficient by its expression level and summing the results. Median values delineated LGG patients into two categories, followed by Kaplan–Meier (KM) survival analysis to evaluate the system. The analysis employed R's “Survival,” “survminer”, and “timeROC” packages to evaluate overall survival and generate ROC curves for 1‐, 3‐, and 5‐year follow‐up within various subgroups. A nomogram was crafted by melding clinical information with risk assessments. The nomogram's reliability was meticulously evaluated through the creation of calibration charts and conducting a multivariate ROC analysis across various follow‐up periods, such as at 1, 3, and 5 years.

### 2.5 LMIRGs Score and Correlation With TMB and Gene Mutations

A statistical examination was conducted to investigate the link between the LMRIGs rating and the TMB metric. Next, The Kaplan–Meier method evaluated distinctions in overall survival among groups with differing TMB values. Subjects were classified into four tiers according to their median tumor mutational burden levels: high TMB and high risk, high TMB and low risk, low TMB and high risk, and low TMB and low risk. Their overall survival (OS) was then compared. TCGA‐hosted somatic mutation information was visualized via a waterfall graph utilizing the “maftools” R package.

### 2.6 Comparative Analysis of TME in Subgroups

The ESTIMATE method was applied to determine the proportions of immune cells (ImmuneScores) and stromal cells (StromalScores). To assess the differences in immune cell infiltration between high‐risk and low‐risk groups, several algorithms were employed, including TIMER, CIBERSORT‐ABS, QUANTISEQ, EPIC, MCPCOUNTER, CIBERSORT, and XCELL.

### 2.7 Gene Set and Pathway Enrichment Study for Differentially Expressed Genes in Risk Stratified Groups

The package of “limma” in R was used to evaluate the differentially expressed genes (DEGs) between the high‐risk and low‐risk groups. Subsequently, Gene Ontology (GO) and Kyoto Encyclopedia of Genes and Genomes (KEGG) analyses were conducted to pinpoint pathways linked to these DEGs. GSEA analysis was executed via clusterProfiler (version 4.0.0), utilizing gene sets sourced from MSigDB. Genes were ranked based on log2 fold change (log2FC) in the analysis, with 1000 permutations performed, and results were considered significant when *p*.adjust < 0.05. GSVA analysis, executed with the GSVA R package, was conducted to ascertain possible variances in biological function among the groups.

### 2.8 Drug Sensitivity Analysis

The Cancer Drug Sensitivity Genomics database was used to predict how sensitive LGG patients are to various chemotherapy drugs. Therefore, the “oncopredict” algorithm was utilized to compute the IC50 values for a range of typical medications, thereby shedding light on their efficacy in modulating particular biological pathways or targets. Box plots depicting the outcomes were created via the R software's “pRRophetic” and “ggplot2” packages. The Wilcoxon rank‐sum test determined variations in medication responsiveness across high‐risk and low‐risk patient subsets.

### 2.9 Human Protein Atlas (HPA) Database Analysis

To verify whether the candidate genes exhibited tissue‐specific expression patterns, researchers sourced immunohistochemical images and corresponding expression profiles from the Human Protein Atlas (HPA) database (available at https://www.proteinatlas.org/). These datasets were then analyzed to identify any notable differences in gene expression between healthy and cancerous tissues.

### 2.10 Cell Culture and Transfection

U118 and LN229 cells were purchased from Promocell, while NHA, U251, and U87 cells were procured from the Chinese Academy of Sciences Cell Bank (Shanghai, China). To culture these cell lines, Dulbecco's Modified Eagle Medium (DMEM) supplemented with 10% fetal bovine serum (FBS) and 1% penicillin/streptomycin solution (Beyotime) was used. The cultures were maintained at 37°C in a CO2‐enriched atmosphere with a 5% CO_2_ concentration. For transfection, glioma cells were cultured in six‐well plates until they reached approximately 60% confluency. A viral solution was then introduced into the culture medium, which was additionally supplemented with 8 μg/mL polybrene (Solarbio, China). Following transfection, puromycin selection (Beyotime, China) was applied to isolate the successfully modified populations. The shRNA system was employed to knock down endogenous PLA2G2A. The shRNA sequences are provided in Table 2.

**TABLE 2:** Primer sequences used in this study.

Primer	Sequences
**ShRNA**	
*PLA2G2A* ShRNA‐1 top strand	GATCCGAGACCCTCCTACTGTTGGCAGTGATCTCG AGATCACTGCCAACAGTAGGAGGGTCTTTTTTTG
*PLA2G2A* ShRNA‐1 bottom strand	AATTCAAAAAAAGACCCTCCTACTGTTGGCAGTGA TCTCGAGATCACTGCCAACAGTAGGAGGGTCTCG
*PLA2G2A* ShRNA‐2 top strand	GATCCGACTGCAGGCCCATGGGAATTTCTC GAGAAATTCCCATGGGCCTGCAGTTTTTTTG
*PLA2G2A* ShRNA‐2 bottom strand	AATTCAAAAAAACTGCAGGCCCATGGGAAT TTCTCGAGAAATTCCCATGGGCCTGCAGTCG
*PLA2G2A* ShRNA‐3 top strand	GATCCGCGTGGATGTGGCACCAAATTTCTC GAGAAATTTGGTGCCACATCCACGTTTTTTG
*PLA2G2A* ShRNA‐3 bottom strand	AATTCAAAAAACGTGGATGTGGCACCAAAT TTCTCGAGAAATTTGGTGCCACATCCACGCG
**RT‐qPCR**	
*PLA2G2A* forward primer	AAGAACCTCCTACTGTTGGC
*PLA2G2A* reverse primer	CCACATCCAAGTTTCTCCAG
*TNFRSF12A* forward primer	CTCTGAGCCTGACCTTCGTG
*TNFRSF12A* reverse primer	GTCTCCTCTATGGGGGTGGT
*VAV3* forward primer	ATTGCCATCGCTCGGTATGACTTC
*VAV3* reverse primer	GCCCACCCTGCCATTTACTTCTC
*ACTB* forward primer	CATGTACGTTGCTATCCAGGC
*ACTB* reverse primer	CTCCTTAATGTCACGCACGAT

### 2.11 Migration and Invasion Assay

Cell migration tests were performed using Transwell chambers, adhering to the manufacturer's guidelines. The cells transfected were seeded in Transwell filters containing serum‐free medium; concurrently, 1 mL of 20% FBS medium was introduced into the lower compartment to serve as a chemotactic agent. Following 24 h of incubation, the cells that migrated to the lower chamber were fixed in 4% paraformaldehyde for 20 min and then stained with crystal violet for 15 min. Following two washes with Phosphate‐Buffered Saline (PBS), images were taken and documented under a microscope.

In accordance with the manufacturer's protocol, liquid Matrigel (Corning) was diluted at a ratio of 1:9 in serum‐free medium and allowed to polymerize at 37°C for 1 h. Subsequently, 70 μL of the Matrigel dilution was introduced into the Transwell inserts within a 24‐well plate, followed by incubation in a cellular culture incubator for 60 min to achieve Matrigel polymerization. Cells were diluted in 200 μL of serumless buffer to reach 5 × 10^4^ cells/mL and then transferred to the upper compartment. 600 μL of complete medium was pipetted into the lower compartment, followed by a 24‐h incubation in a cell culture chamber. The chamber was extracted, the medium aspirated, and the cells subjected to two washes in PBS. A swab was applied to dislodge Matrigel and any cells from the upper chamber. Then, 600 μL of 4% paraformaldehyde was added to the lower chamber for 20 min. After removing the 4% paraformaldehyde, 600 μL of crystal violet was added to the lower chamber for staining for 15 min. Images were captured post‐duplicate PBS washes via a microscope.

### 2.12 Tissue Samples

Eight tumor specimens and their corresponding non‐cancerous neighboring tissue samples from LGG patients were gathered for this research. Patients received surgical excision at Ningbo University's First Affiliated Hospital (Ningbo, China). None of the patients had undergone radiotherapy or chemotherapy before surgery. After resection, all tissue samples were quickly snap‐frozen in liquid nitrogen and stored at −80°C until RNA extraction. Participants granted informed consent, and the research was authorized by Ningbo University First Affiliated Hospital's Ethics Committee (approval ID: 2023127A). Clinical parameters are shown in Table 3.

**TABLE 3:** Clinical data of patients in this study.

Patient ID	Sex	Age (years)	Tumor location	Pathology type	WHO grade	Treatment
1	Male	45	Frontal lobe	Astrocytoma	III	Surgery + chemoradiotherapy
2	Male	65	Frontal lobe	Oligodendroglioma	III	Surgery only
3	Female	29	Frontal lobe	Astrocytoma	II	Surgery only
4	Male	48	Parietal lobe	Oligodendroglioma	II	Surgery + chemotherapy
5	Female	40	Frontal lobe	Oligodendroglioma	II	Surgery only
6	Female	36	Frontal lobe	Oligodendroglioma	II	Surgery only
7	Female	35	Frontal lobe	Oligodendroglioma	II	Surgery + chemoradiotherapy
8	Female	57	Frontal lobe	Oligodendroglioma	II	Surgery + chemoradiotherapy

### 2.13 RT‐qPCR Detection

RNA extracted from glioma and neighboring normal tissue samples with TRIzol solution (Invitrogen, Carlsbad, CA, USA). cDNA was generated via reverse transcription from RNA with the TransGen Biotech High‐Capacity Reverse Transcription Kit. We conducted quantitative polymerase chain reaction (qPCR) using the LightCycler 480 system from Roche, located in Mannheim, Germany, and the SYBR Green SuperMix from TransGen Biotech, based in Beijing, China. The primers for the qPCR were meticulously crafted with the aid of Primer 6 software, developed by Premier Biosoft in California, USA. The primer sequences are shown in Table 3.

### 2.14 Western Blot

Tumor tissue lysates from beneath the skin were prepared using RIPA lysis buffer (Thermo Fisher Scientific) along with a protease inhibitor cocktail (Roche Diagnostics). Samples' protein levels were evaluated using the Beyotime BCA kit (China). Subsequently, they were electrophoretically separated via SDS‐PAGE before being transferred to a PVDF membrane. To block nonspecific sites, the membrane was incubated with 5% milk for 1 h at room temperature, after which the relevant primary antibody was applied and left to incubate overnight at 4°C. After washing the membrane three times for 30 min with PBS with Tween‐20 (PBST), it was incubated for 1 h with the secondary antibody. The membrane was then washed again three times with PBST and visualized using chemiluminescence. The antibodies listed below were employed in the experiments: β‐actin (1:20000, Affinity, Cat: AF7018), PLA2G2A (1:1000, ABclonal, Cat: A19252), E‐Cadherin (1:1000, Santa Cruz, Cat: sc‐8426), Vimentin (1:1000, ABclonal, Cat: A19607), PCNA (1:1000, Santa Cruz, Cat: sc‐53 407), H1F‐1α (1: 1000, Proteintech, Cat: 20960‐1‐AP), LDHA (1:1000, Proteintech, Cat: 19987‐1‐AP), MMP2 (1:3000, ABclonal, Cat: A6247) and MMP9 (1:1000, Santa Cruz, Cat: sc‐393 859).

### 2.15 Cell Counting Kit‐8 (CCK8)

U251 and LN229 glioma cells, infected with Sh‐NC and Sh‐*PLA2G2A* (1000 cells per well in 100 μL of DMEM medium), were seeded into 96‐well tissue culture plates, with three replicates for each group. At six time points (0, 24, 48, 72, 96, and 120 h), 10 μL of CCK8 solution (Selleck Chemicals) was added to each well, followed by incubation at 37°C for 2 h. After gently shaking the plate for 1 min, the absorbance of each sample was measured at 450 nm using an ELISA analyzer.

### 2.16 Lactate Level Measurement

To assess the effect of *PLA2G2A* knockdown on lactate levels, lactate concentrations were measured using the Chekine Lactate Assay Kit (Chekine, Cat: KTB1100) according to the manufacturer's instructions. Supernatants from U251 cells and *PLA2G2A* knockdown U251 cells were collected and reacted with the lactate reagent. Lactate concentrations were determined by colorimetric analysis.

### 2.17 Xenograft Tumor Model

This study complied with all relevant ethical standards for animal experimentation and was approved by the Animal Care and Use Committee of Ningbo University (No. 2023127A). U251 cells stably expressing shRNA‐*PLA2G2A* or the control empty vector (4.0 × 10^6^ cells/200 μL per mouse) were subcutaneously implanted into the axillary region of 4‐week‐old female BALB/c nu/nu nude mice (*n* = 6 per group). The animals were kept in standard laboratory conditions with a 12‐h light/dark cycle, a temperature range of 22°C–25°C, and humidity levels between 40% and 60%, with free access to food and water. Tumor size was calculated using the formula: Volume = Length × Width^2^/2. The experiment was conducted over a 20‐day period, and no animal deaths were recorded during the study. Animal health was assessed daily, and tumor size was recorded every 4 days. Six mice were used per group in total. After 20 days, the mice were euthanized, and tumor samples were excised, weighed, and processed for protein extraction prior to Western blotting.

### 2.18 Statistical Analysis

Data exploration and graphical representation were executed using R version 4.4.1. For all in vitro experiments, unless stated otherwise, at least three independent biological samples were used. Statistical comparisons were conducted using appropriate methods based on variable types. For categorical data, we performed Chi‐square analyses to evaluate differences between groups. Continuous variables were analyzed either with Wilcoxon rank‐sum tests (for non‐parametric distributions) or *t*‐tests (for normally distributed data). When examining relationships between continuous measures, we calculated Pearson's *r* to quantify linear associations. To ensure the robustness of our findings, we conducted a correlation analysis, applying the Bonferroni correction to bolster the credibility of the outcomes. For comparing survival rates across various subgroups, we relied on Kaplan–Meier survival analysis, supplemented by log‐rank tests. Data analysis was performed using GraphPad Prism 9.0 for Windows (GraphPad Software Inc.). A *p*‐value of below 0.05 was considered statistically significant.

## 3 Results

### 3.1 Identification of Lactate‐Related Gene Clusters in LGG

**FIGURE 1:**
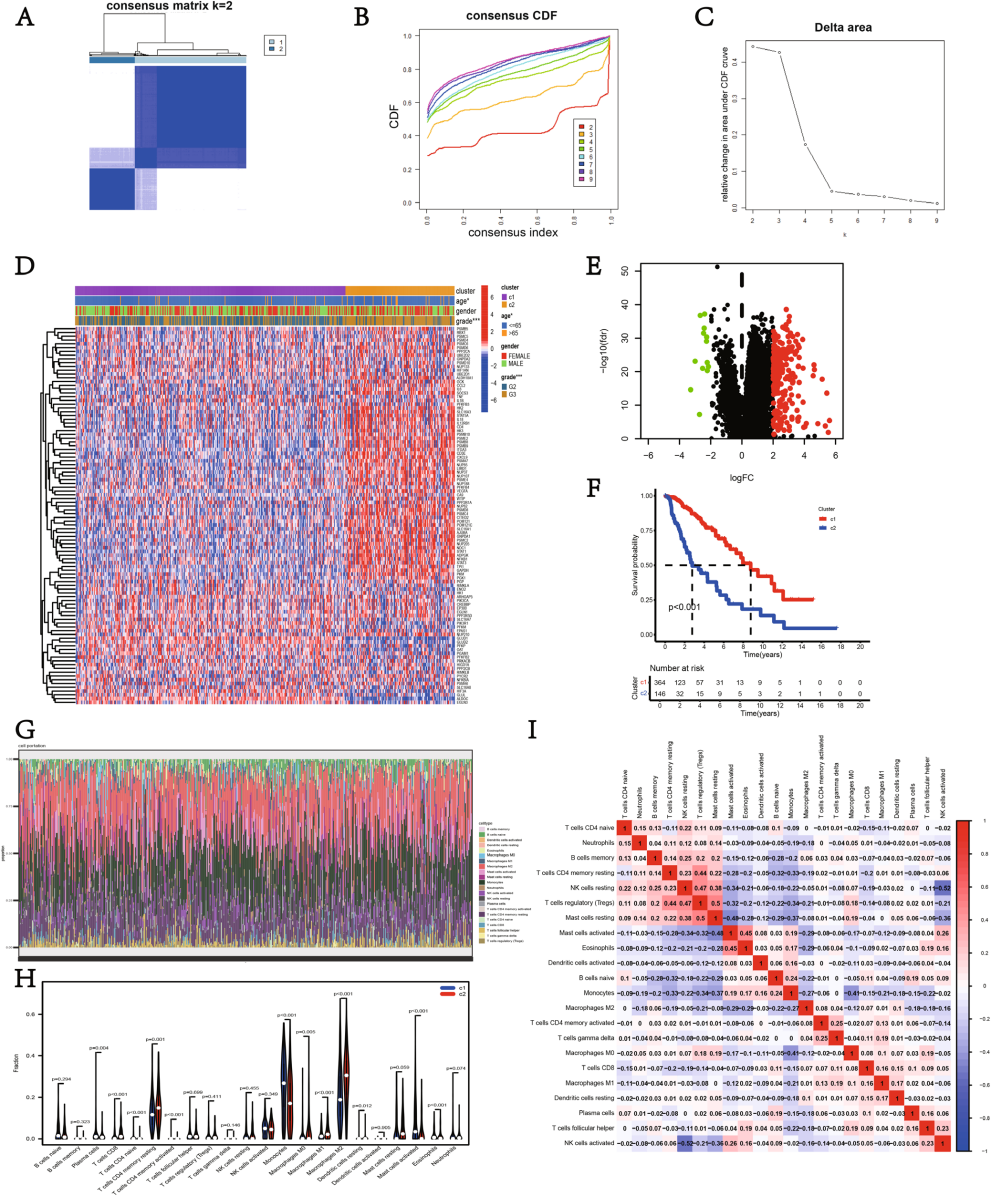
Molecular subtyping, differential gene expression, and immune infiltration analysis. (A–C) Cluster analysis indicates a stable separation of samples into two distinct groups (denoted as C1 and C2). (D) The heatmap shows significant differences in the differential gene expression profiles between the two sample groups. (E) The volcano plot displays the upregulated (red) and downregulated (green) differential genes. (F) Kaplan–Meier survival curves indicate significant differences in overall survival between the two subtypes. (G) The CIBERSORT algorithm shows the immune cell composition in different samples. (H) The violin diagram showcases the disparities in the prevalence of particular immune cell categories across the two groups being analyzed. (I) The correlation heat map displays the interconnectedness of various immune cell subsets.

**FIGURE 2:**
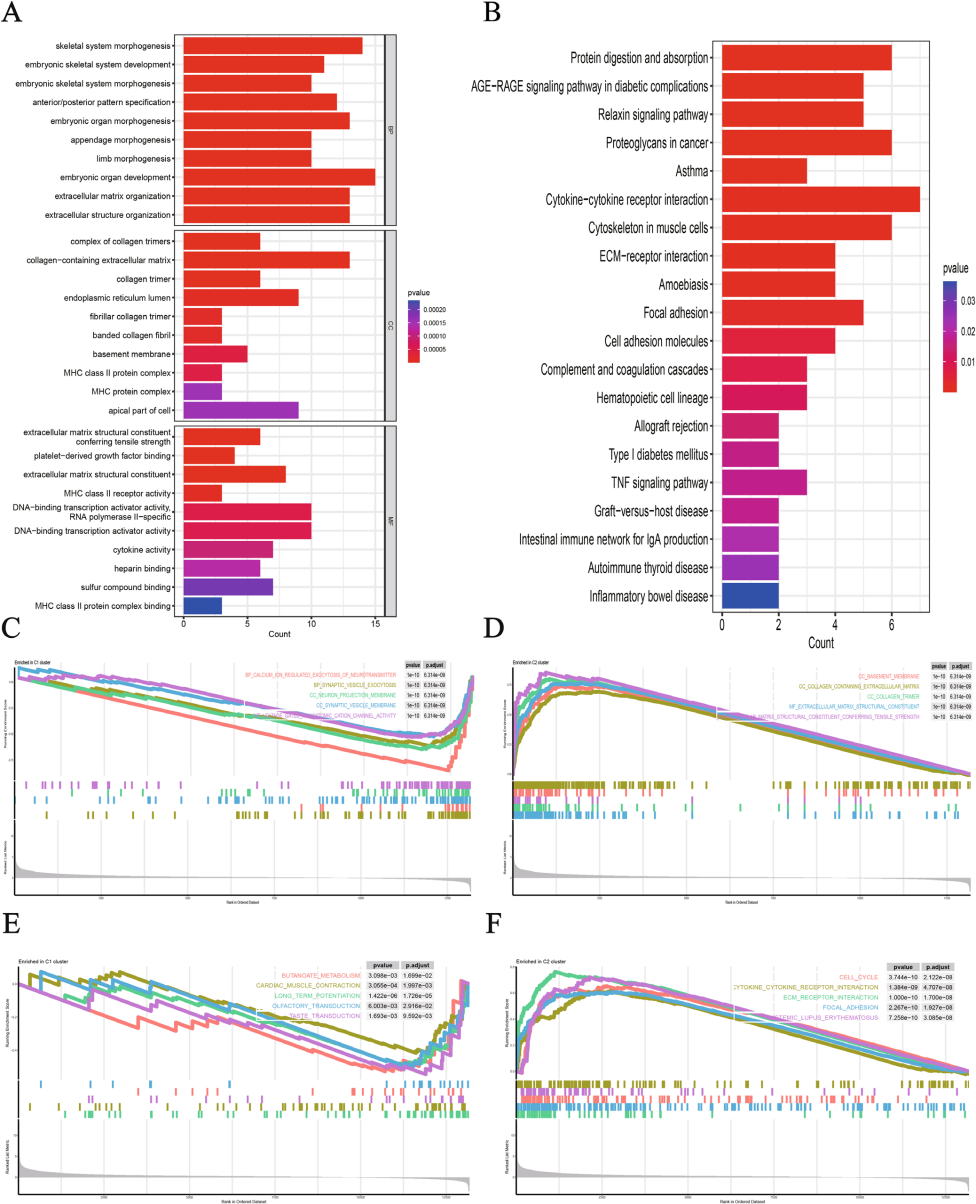
Differential gene functional enrichment analysis. (A) GO enrichment results. (B) KEGG pathway analysis. (C–F) GSEA shows that DEGs are mainly involved in ECM remodeling, immune‐inflammatory regulation, and tumor‐related pathways.

Figure 1 depicts the application of consensus clustering in investigating the link between lactate‐associated gene expression and LGG. According to the CDF curve, patients were divided into two groups (C1 and C2) (Figure 1A–C). Figure 1D depicts the interplay among lactate‐associated gene groups, patient characteristics, and patterns of genetic expression. Kaplan–Meier survival analysis revealed a significant difference in overall survival (OS) between Cluster 1 and Cluster 2 (*p* < 0.05), indicating that this classification may have prognostic significance (Figure 1F). Given the close relationship between immune cell variation and LGG progression, we further assessed the immune cell levels among the two patient groups (Figure 1G). The results showed that, relative to C2, C1 exhibited greater numbers of plasma cells, monocytes, eosinophils, and activated mast cells. In contrast, C1 had noticeably lower levels of CD8+ T cells, naive CD4+ T cells, resting and activated CD4+ T cells, M0/M1/M2 macrophages, resting dendritic cells, and resting mast cells (Figure 1H). The immune cell correlation heatmap revealed a complex immune cell network, such as the positive correlation between CD4+ T cells and M1 macrophages, and the negative correlation between NK cells and plasma cells (Figure 1I). GO enrichment analysis suggests that differentially expressed genes (DEGs) are predominantly significantly associated with biological processes such as skeletal system morphogenesis, embryonic organ development, and extracellular matrix (ECM) organization. The differentially expressed genes (DEGs) were predominantly associated with extracellular matrix components containing collagen, basement membrane structures, and major histocompatibility complex (MHC) protein assemblies. From a molecular function perspective, these DEGs demonstrated notable enrichment in MHC binding interactions, cytokine‐related activities, and DNA‐binding functions of transcription factors (Figure 2A). KEGG pathway analysis further showed that these genes were primarily associated with immune and inflammation‐related pathways, such as the AGE–RAGE signaling pathway, cytokine‐receptor interactions, cell adhesion molecules, transplant rejection, type I diabetes, and inflammatory bowel disease, among others as well as cancer‐related pathways (such as proteoglycans in cancer, TNF signaling pathway) (Figure 2B). Gene set enrichment analysis (GSEA) further confirmed these findings, indicating that differentially expressed genes were notably enriched in ECM‐receptor interactions, immune regulation, and TGF‐β signaling pathways (Figure 2C); cell adhesion and cytoskeleton remodeling (Figure 2D); cancer and cell proliferation‐related pathways (Figure 2E); and cytokine‐receptor interactions and inflammatory responses (Figure 2F). To wrap things up, the genes that are expressed differently seem to play a pivotal role in ECM reorganization, as well as in modulating the immune system and inflammation. These genes could very well be intricately tied to the development and advancement of cancer.

### 3.2 Development of the LMIRGs Signature in LGG and Validation of Its Prognostic Value

**FIGURE 3:**
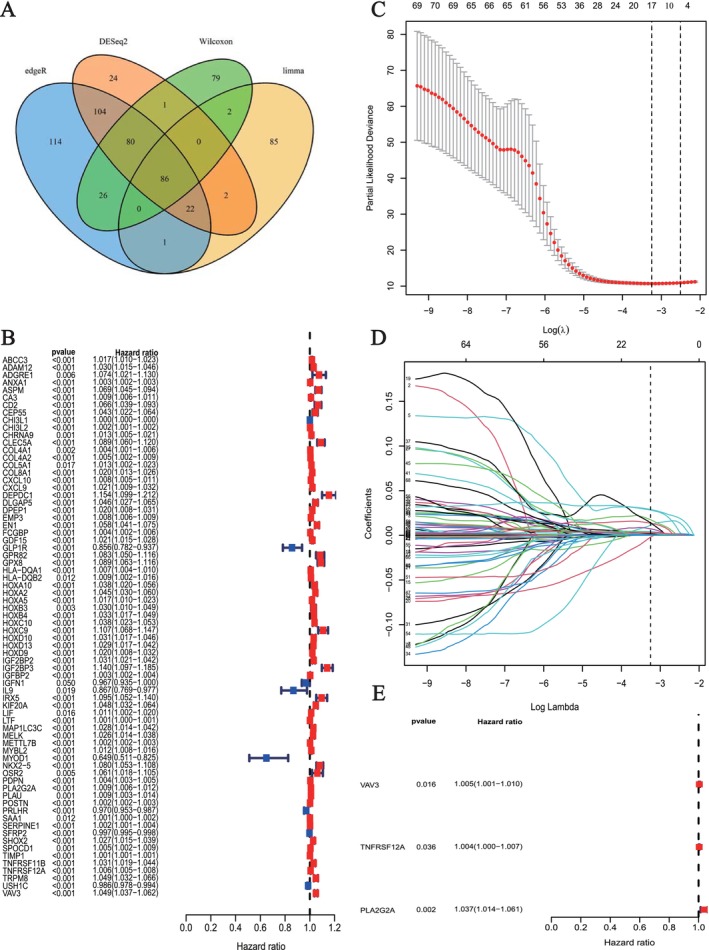
Differential gene screening and prognostic model construction. (A) The Venn diagram shows the gene intersection obtained from four differential gene screening methods (edgeR, DESeq2, Wilcoxon, and limma), with 86 common differential genes selected. (B) Univariate Cox regression identifies significant gene‐survival correlations, with hazard ratios (HR) and *p*‐values illustrated in the forest plot. (C) LASSO regression cross‐validation determines the optimal λ value, with red dots representing the mean error of cross‐validation and gray bars indicating the standard deviation. (D) The LASSO regression path plot shows the trend of regression coefficients as λ values change, with coefficients gradually shrinking to zero and ultimately retaining a few key genes. (E) Multivariate Cox regression analysis selected three key genes: *VAV3, TNFRSF12A,* and *PLA2G2A*, showing their hazard ratios (HR) and *p*‐values.

**FIGURE 4:**
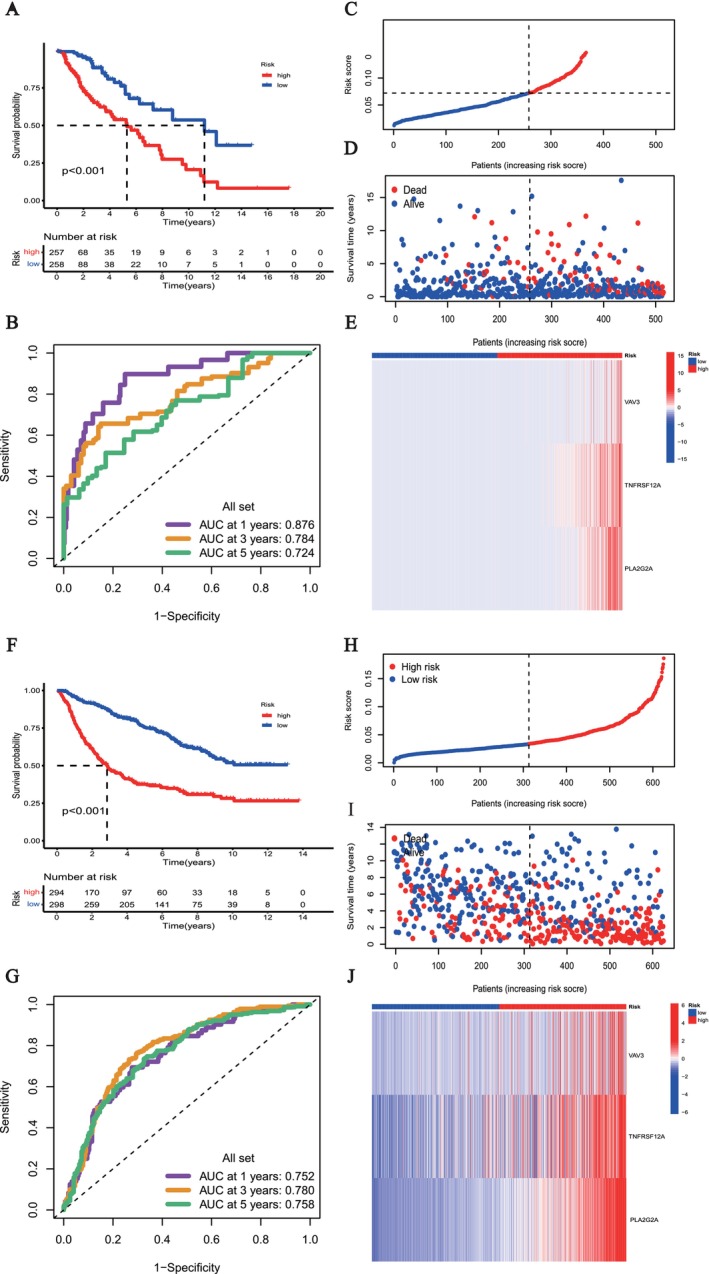
Construction and validation of the LMRIGs prognostic signature in LGG. (A) Distribution of survival status between high‐ and low‐risk patients. (B) ROC curves of the LMRIGs signature at 1, 3, and 5 years to evaluate predictive accuracy. (C) LGG patients stratified into high‐ and low‐risk groups based on risk scores. (D) Expression patterns of risk genes in different subgroups. (E) Heatmap of the expression of three LMRIGs genes (*VAV3, TNFRSF12A*, and *PLA2G2A*). (F) Kaplan–Meier survival analysis in the CGGA cohort, showing better prognosis in the low‐risk group. (G) ROC curves at 1, 3, and 5 years in the CGGA cohort. (H) Risk group classification in the CGGA validation cohort. (I) Expression patterns of risk genes in different subgroups. (J) Expression of risk genes in different subgroups of the CGGA cohort.

Through screening with the “limma”, “edgeR,” “DESeq2” and “Wilcoxon” methods, 86 overlapping genes with significant differential expression were found (FDR < 0.05, |logFC| > 2) (Figure 3A). The univariate Cox regression findings revealed a distinct correlation between particular genes and patient survival rates, with certain genes increasing risk (HR > 1) while others appeared to have a protective effect (HR < 1), as illustrated in Figure 3B. After LASSO and multivariate Cox regression [22], a three‐gene signature, including vav guanine nucleotide exchange factor 3 (*VAV3*), tumor necrosis factor receptor superfamily member 12A (*TNFRSF12A*), and phospholipase A2 group IIA (*PLA2G2A*) (the LMIRGs signature) was developed (Figure 3C–E). The risk score calculation was defined as: Risk score = *VAV3* × 0.0289 + *TNFRSF12A* × 0.0267 + *PLA2G2A* × 0.0326. Kaplan–Meier survival analysis in the TCGA‐LGG cohort showed that patients in the high‐risk group had significantly worse overall survival than those in the low‐risk group (Figure 4A). Time‐dependent ROC curve analysis further demonstrated the strong predictive performance of the risk model, with AUC values of 0.876, 0.784, and 0.724 at 1, 3, and 5 years, respectively (Figure 4B). Based on the calculated risk scores, LGG patients were clearly stratified into high‐ and low‐risk groups using the optimal cutoff value (Figure 4C). The distribution of survival status indicated that mortality increased progressively with higher risk scores, whereas patients with lower risk scores were more likely to survive (Figure 4D). Heatmap analysis revealed distinct expression patterns of the signature genes *PLA2G2A, TNFRSF12A,* and *VAV3* between the two risk groups, supporting the biological basis of the risk model (Figure 4E). These findings were consistently validated in the CGGA cohort, where Kaplan–Meier analysis again showed significantly poorer survival in the high‐risk group compared with the low‐risk group (Figure 4F). The ROC curves in the CGGA dataset confirmed the robustness of the model, yielding AUCs of 0.752, 0.780, and 0.758 at 1, 3, and 5 years, respectively (Figure 4G). Similarly, patients in the CGGA cohort were effectively divided into high‐ and low‐risk categories according to the risk score distribution (Figure 4H), with higher risk scores associated with increased mortality (Figure 4I). Consistent gene expression differences between risk groups were also observed in the CGGA cohort, further validating the stability and generalizability of the prognostic signature (Figure 4J).

### 3.3 Construction and Evaluation of the LGG Nomogram Model

**FIGURE 5:**
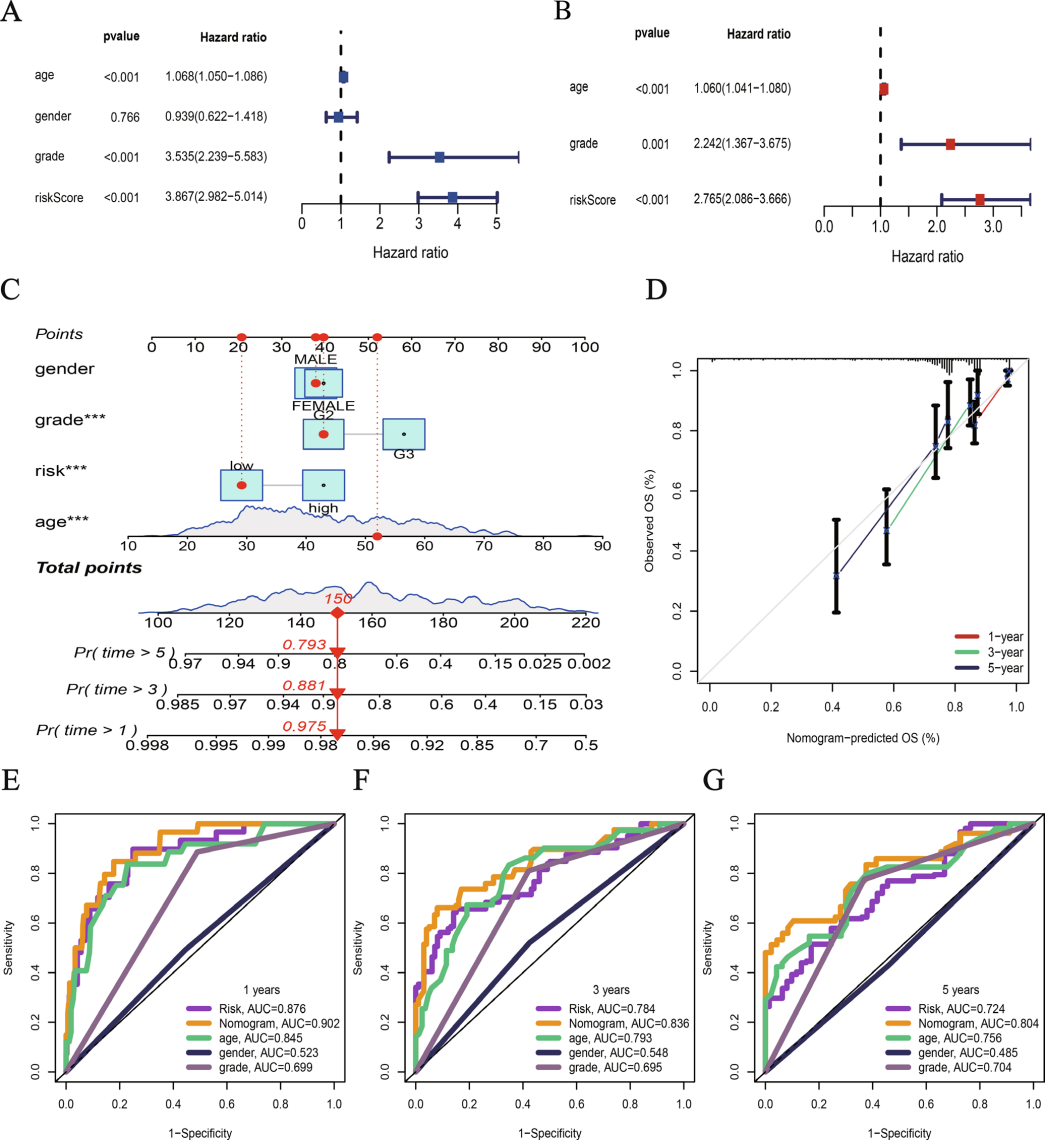
LGG nomogram based on the LMRG signature and clinical features. (A) Univariate Cox regression analysis highlights the correlation between clinical metrics and survival‐related risk scores. (B) A multivariate Cox regression analysis suggests that both risk score and grade act as distinct predictors of prognosis. (C) Combining age, gender, grade, and risk score allows for the development of a nomogram model, which can forecast 1‐, 3‐, and 5‐year overall survival probabilities. (D) Calibration curves show high consistency between the predicted survival rates of the nomogram and actual survival rates. (E–G) ROC graphs evaluate the predictive accuracy of the risk metric and nomogram for 1‐, 3‐, and 5‐year survival rates.

Both univariate and multivariate Cox regression analyses indicated that age, grade, and risk score were strongly linked to survival. In multivariate analysis, both risk score and grade independently predicted outcomes, with risk score showing the strongest association (HR = 2.766, 95% CI: 2.068–3.666) (Figure 5A,B). we created a visual predictive tool—a nomogram—that integrates age, sex, tumor grade, and risk assessment to estimate a patient's likelihood of survival at 1, 3, and 5 years (Figure 5C). The model's accuracy was confirmed by calibration plots, which showed near‐perfect alignment between projected results and real‐world data (Figure 5D). The ROC analysis showed that the nomogram model outshone the individual risk score in terms of predictive prowess over various time periods, as evidenced in Figure 5E–G.

### 3.4 Differences in Immune Microenvironment Between High‐ and Low‐Risk Groups

**FIGURE 6:**
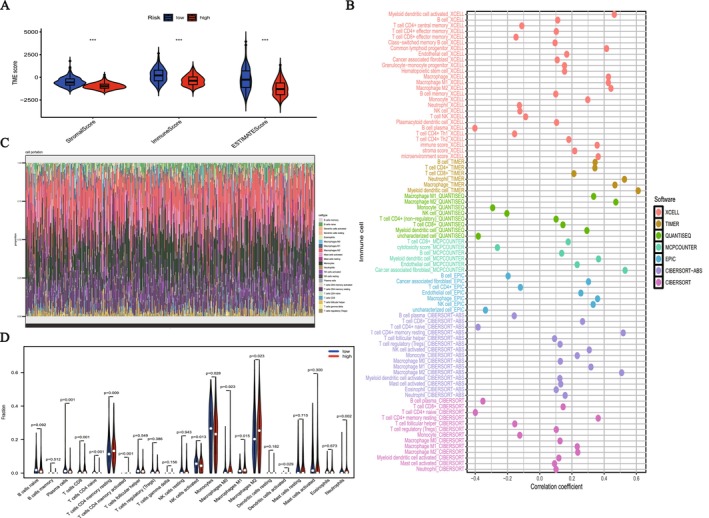
Comparison of tumor microenvironment (TME) characteristics between two groups. (A) The high‐risk cohort demonstrated markedly reduced StromalScore, ImmuneScore, and ESTIMATEScore values when benchmarked against their low‐risk counterparts. (B) Disparities in immune cell infiltration patterns emerged between the risk groups across various computational assessment methods. (C, D) Analysis of immune cell subtypes revealed greater infiltration of T cells, B cells, and dendritic cells in the low‐risk group, whereas the high‐risk group displayed comparatively diminished immune cell presence.

Immune scores and risk scores were compared, revealing significant differences between the high‐risk (red) and low‐risk (blue) groups in StromalScore, ImmuneScore, and ESTIMATEScore (Figure 6A). The high‐risk group showed lower scores in all three immune‐related scores (*p* < 0.001), indicating a poorer immune microenvironment in this group. Specifically, a low StromalScore indicates a weaker stromal immune response, a low ImmuneScore suggests less immune cell infiltration, and a lower ESTIMATEScore reflects an inefficient overall immune microenvironment. Immune cells play a vital role in the tumor microenvironment (TME), influencing cancer progression. The disparity in immune cell levels across the two risk categories was analyzed through a suite of methodologies, which included CIBERSORT, MCPCOUNTER, QUANTISEQ, EPIC, TIMER, CIBERSORT‐ABS and XCELL. Notably, within the high‐risk cohort, there was a marked decrease in the presence of different immune cell types, such as dendritic cells, B cells and both CD4+ and CD8+ T cells. On the flip side, the low‐risk group exhibited a more robust infiltration of immune cells, especially T cells, dendritic cells and B cells, as depicted in Figure 6B. Figure 6C,D show the makeup of immune cell subpopulations in the high‐risk and low‐risk cohorts, revealing that immune cell infiltration is markedly reduced in the high‐risk group relative to the low‐risk group (*p* < 0.05). The low‐risk group exhibited greater immune cell infiltration (e.g., CD8+ T cells, dendritic cells, B cells), while the high‐risk group showed relatively less immune cell infiltration. The analysis revealed a striking correlation between immune cell presence and risk stratification. Patients classified as low‐risk demonstrated heightened immune infiltration, pointing to a robust and advantageous tumor microenvironment. On the other hand, those in the high‐risk group showed markedly reduced immune cell activity—a telltale sign of possible immune suppression mechanisms that might accelerate cancer progression and predict poorer clinical outcomes.

### 3.5 LMIRGs Features and Tumor Mutation Burden Analysis

**FIGURE 7:**
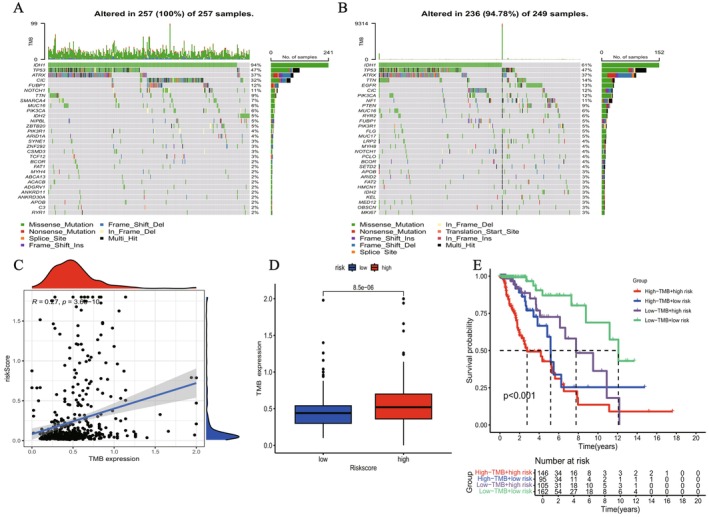
Relationship between LMRIGs signature, gene mutations, and tumor mutation burden (TMB). (A, B) Mutation profiles of the top 20 high‐frequency mutated genes in different subgroups. (C, D) Linear regression reveals a substantial positive link between the risk metric and TMB. (E) Kaplan–Meier survival analysis combining TMB and risk score shows that patients in the high TMB and high‐risk group have the worst prognosis, while those in the low TMB and low‐risk group have the best prognosis.

Figure 7A,B present the top 20 genes with mutations observed across the subgroups. The results indicate that samples with high TMB are generally accompanied by more gene mutations, suggesting a robust link between TMB and mutational load. The analysis via linear regression showed that, unlike the low‐risk cohort, the high‐risk group exhibited higher TMB ratings, which underscored a robust positive link between TMB and the risk assessment (see Figure 7C,D). In Kaplan–Meier analysis, we assessed how TMB and risk score integration influenced patient survival (Figure 7E). Survival curves for the four groups demonstrated significant differences in survival (*p* < 0.001). These findings indicate that the combined use of TMB and risk score provides a more accurate prediction of patient survival. A high TMB combined with a high‐risk score is strongly linked to a worse prognosis, whereas a low TMB coupled with a low‐risk score correlates with a more favorable prognosis.

### 3.6 Functional Enrichment of the LMIRGs Signature

**FIGURE 8:**
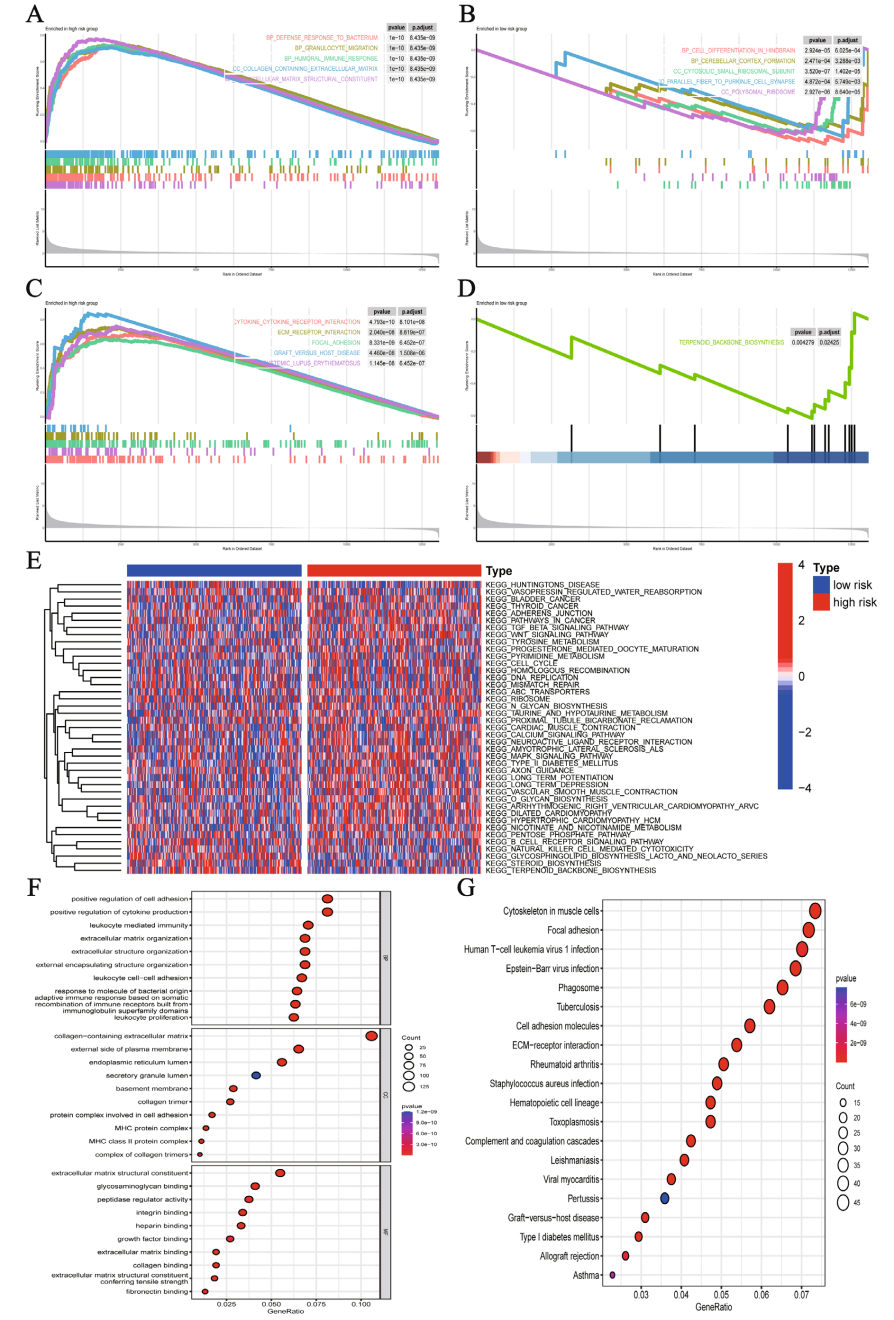
Functional enrichment analysis between high‐ and low‐risk groups. (A) The high‐risk cohort shows significant activation of pathways linked to immune response and inflammatory processes. (B) The low‐risk group exhibits enrichment in biological pathways associated with cellular differentiation and developmental regulation. (C) The high‐risk category demonstrates a strong connection to pathways implicated in immune‐related disorders. (D) The low‐risk group is enriched in metabolism‐related pathways. (E) The GSVA heatmap shows stronger immune activity in the high‐risk group and higher metabolic activity in the low‐risk group. (F) GO analysis shows that the differential genes are involved in adhesion, ECM organization, and immune regulation. (G) KEGG analysis indicates significant enrichment in cytoskeleton organization, ECM–receptor interaction, complement/coagulation cascades, viral infection pathways, and immune disease‐related pathways.

After performing GSEA and GSVA among risk subgroups, we detected notable pathway variations. Figure 8A illustrates that the high‐risk group is predominantly associated with immune response pathways, including defense response to bacterium, granulocyte migration, and collagen‐containing extracellular matrix. The results demonstrate that individuals in the high‐risk category exhibit heightened immune system activity and inflammatory responses. As illustrated in Figure 8B, the low‐risk group displays distinct biological patterns, with significant pathway enrichment related to cellular differentiation, bone development, and the regulation of cell polarity. These processes are closely linked to tissue homeostasis and the maintenance of structural functions, suggesting that the biological traits of the low‐risk group are more aligned with tissue development and differentiation. As shown in Figure 8C, the high‐risk cohort demonstrates a pronounced overrepresentation of immune‐linked pathological pathways. These include cytokine‐receptor signaling, graft‐versus‐host reactions, lupus erythematosus, and transplant rejection mechanisms—all pointing to significant immune dysregulation. These pathways imply that the high‐risk group may experience enhanced immune dysregulation and inflammatory responses [23]. Figure 8D demonstrates that the low‐risk group is particularly associated with metabolism‐related pathways, with terpenoid backbone biosynthesis being the most prominent pathway. This further implies that the biological traits of the low‐risk group are more focused on metabolic processes than on immune hyperactivation. Figure 8E displays the heatmap from GSVA analysis, highlighting variations in various signaling pathways across the risk groups. It is evident that the high‐risk group shows greater enrichment in immune and inflammation‐related pathways, whereas the low‐risk group is more prominent in metabolism‐related pathways. This finding aligns closely with the results from GSEA. A functional enrichment analysis of differential genes was then conducted. GO analysis (Figure 8F) revealed that these genes are primarily associated with processes like cell adhesion, extracellular matrix organization, and immune response modulation. KEGG analysis (Figure 8G) revealed significant enrichment of differentially expressed genes in pathways associated with the cytoskeleton, ECM‐receptor interactions, complement and coagulation cascades, viral infections (such as EB virus, HIV, HTLV‐1 infection), and various immune‐related diseases (including rheumatoid arthritis, asthma, graft‐versus‐host disease). To sum up, individuals in the high‐risk category predominantly exhibit overactive immune signaling and heightened inflammatory reactions. In contrast, those in the low‐risk group tend to display stronger ties to cell differentiation, tissue development, and metabolic processes.

### 3.7 Analysis of the Correlation Between Drug Sensitivity and Risk Score

**FIGURE 9:**
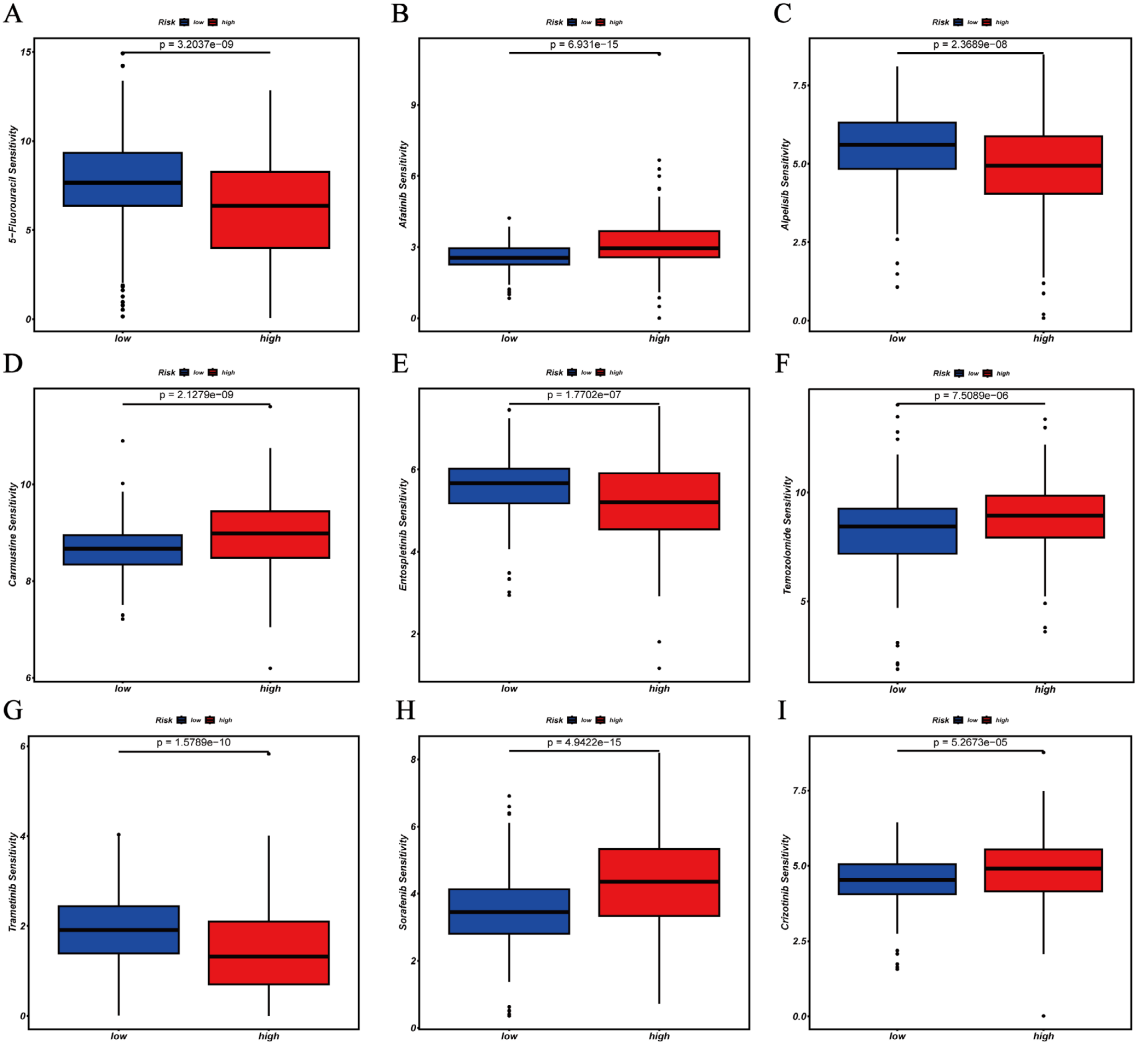
Drug sensitivity analysis between two groups. (A,C,E,G) The members of the low‐risk category are particularly susceptible to the effects of 5‐Fluorouracil, Alpelisib, Entospletinib, and Trametinib. (B,D,F,H,I) Those in the high‐risk category are more likely to react to Afatinib, Carmustine, Temozolomide, Sorafenib, and Crizotinib.

To assess potential variations in drug sensitivity between the low‐risk and high‐risk groups, we analyzed the relationship between LGG patients' risk scores and the IC50 values of chemotherapy and targeted therapy drugs. Significant differences in chemotherapy drug response were observed across patient risk groups (Figure 9). The low‐risk group was more sensitive to drugs such as 5‐Fluorouracil, Alpelisib, Entospletinib, and Trametinib, while the high‐risk group showed higher sensitivity to drugs like Afatinib, Carmustine, Temozolomide, Sorafenib, and Crizotinib. This indicates that the risk classification reflects not only differences in prognosis but could also serve as a reference for tailoring personalized clinical treatments. The high‐risk group may respond better to targeted therapies and platinum‐based chemotherapy, while the low‐risk group might be more appropriate for treatments based on fluoropyrimidine and hormone therapies.

### 3.8 Expression Characteristics of *VAV3* , *TNFRSF12A* and *PLA2G2A* and Experimental Validation

**FIGURE 10:**
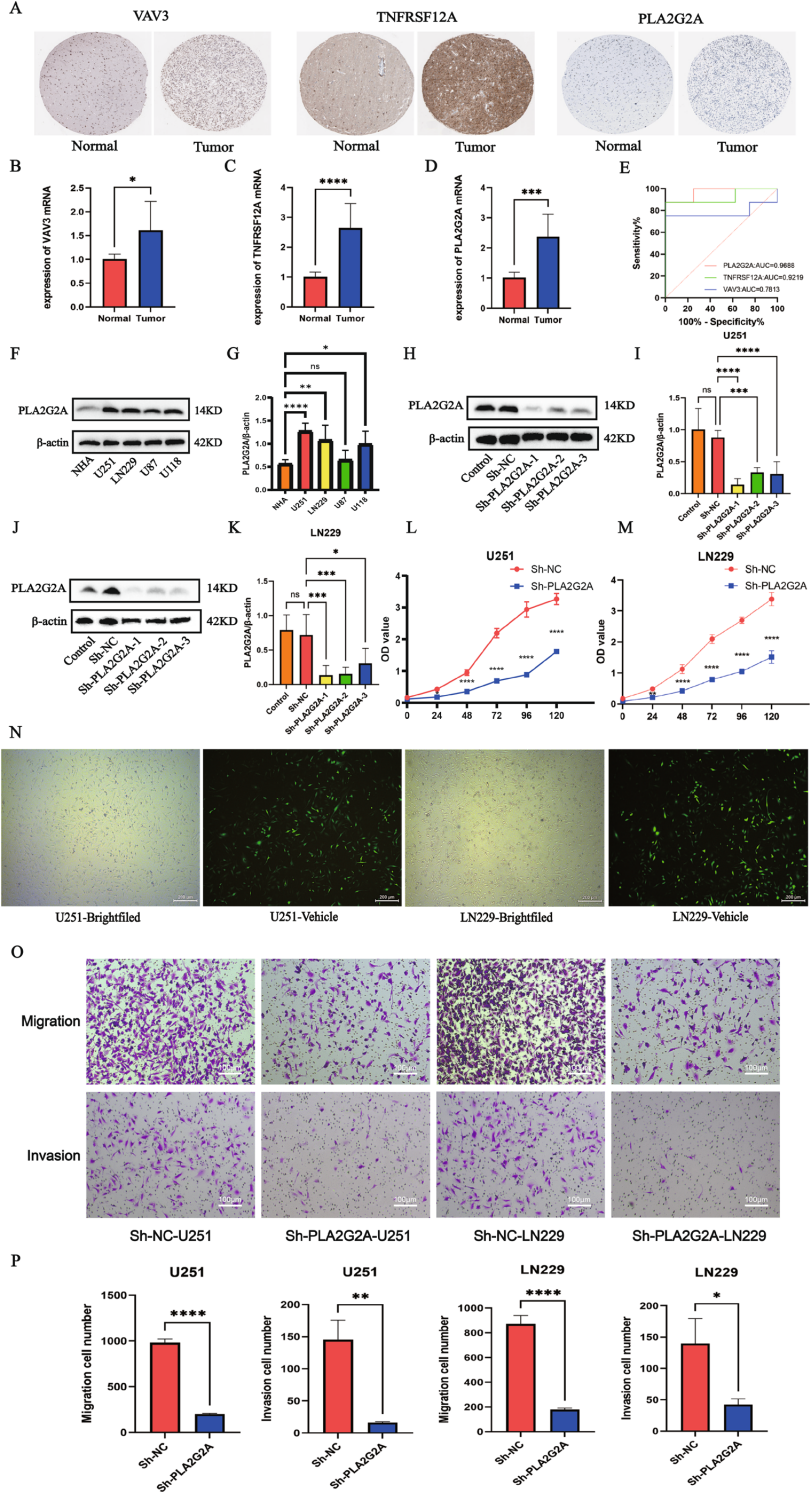
Expression validation of key model genes and in vitro functional experiments of PLA2G2A. (A) Immunohistochemical results from the Human Protein Atlas (HPA) database show that the expression levels of VAV3, TNFRSF12A, and PLA2G2A are significantly higher in tumor tissues than in normal tissues. (B–D) qRT‐PCR results from clinical samples show that the mRNA levels of *VAV3, TNFRSF12A*, and *PLA2G2A* are significantly elevated in tumor tissues (*p < 0.05, ***p < 0.001, ****p < 0.0001). (E) Receiver operating characteristic curves (ROC) of *PLA2G2A* mRNA, *TNFRSF12A* mRNA, and *VAV3* mRNA in subjects. (F, G) Western blot analysis of PLA2G2A protein expression in different glioma cell lines (*p < 0.05, **p < 0.01, ****p < 0.0001). (H, I) After PLA2G2A knockdown in U251 cells using shRNA, Western blot confirmed transfection efficiency (***p < 0.001, ****p < 0.0001). (J, K) After PLA2G2A knockdown in LN229 cells using shRNA, Western blot confirmed transfection efficiency (*p < 0.05, ***p < 0.001). (L, M) CCK‐8 assay results show that PLA2G2A downregulation significantly inhibits the proliferative ability of U251 and LN229 glioma cells (*p < 0.05, **p < 0.01, ****p < 0.0001) (N) Immunofluorescence detection shows successful construction of stable U251 and LN229 transfected cells. (O) Transwell assay results show that PLA2G2A knockdown significantly reduces the migration and invasion abilities of U251 and LN229 tumor cells. (P) Quantitative analysis further confirms that the number of migrating and invading cells in the sh‐PLA2G2A treatment group is significantly lower than in the control group (*p < 0.05, **p < 0.01, ****p < 0.0001).

To further confirm the expression patterns and possible biological roles of the candidate genes in tumor tissues, we first analyzed the immunohistochemistry results of VAV3, TNFRSF12A, and PLA2G2A using the HPA (Human Protein Atlas) database. Figure 10A depicts significantly elevated expression levels of VAV3, TNFRSF12A, and PLA2G2A in cancerous tissue versus healthy tissue. Specifically, VAV3 staining was primarily localized to the cell membrane and cytoplasm, TNFRSF12A was widely distributed in tumor cells with high staining intensity, and PLA2G2A showed specific positive expression in tumor tissues, with almost no staining in normal tissues, indicating its potential key role in tumorigenesis and development. In order to bolster the credibility of our database results, we subjected clinical samples to quantitative real‐time PCR analysis. This revealed a substantial increase in the mRNA expression of *VAV3*, *TNFRSF12A* and *PLA2G2A* in tumor tissue, a stark contrast to the levels observed in neighboring healthy tissue (Figure 10B–D). To further evaluate the diagnostic potential of *PLA2G2A*, *TNFRSF12A* and *VAV3* mRNA expression levels in predicting LGG, we conducted a Receiver Operating Characteristic (ROC) curve analysis. The results were striking: *PLA2G2A* demonstrated exceptional predictive accuracy with an AUC of 0.9688, while *TNFRSF12A* also showed strong diagnostic capability at 0.9219. *VAV3*, though less robust, still offered meaningful predictive value with an AUC of 0.7813. These findings underscore the clinical relevance of these biomarkers in LGG prognosis (Figure 10E).

### 3.9 Investigating the Functional Role of PLA2G2A in Tumor Cells

**FIGURE 11:**
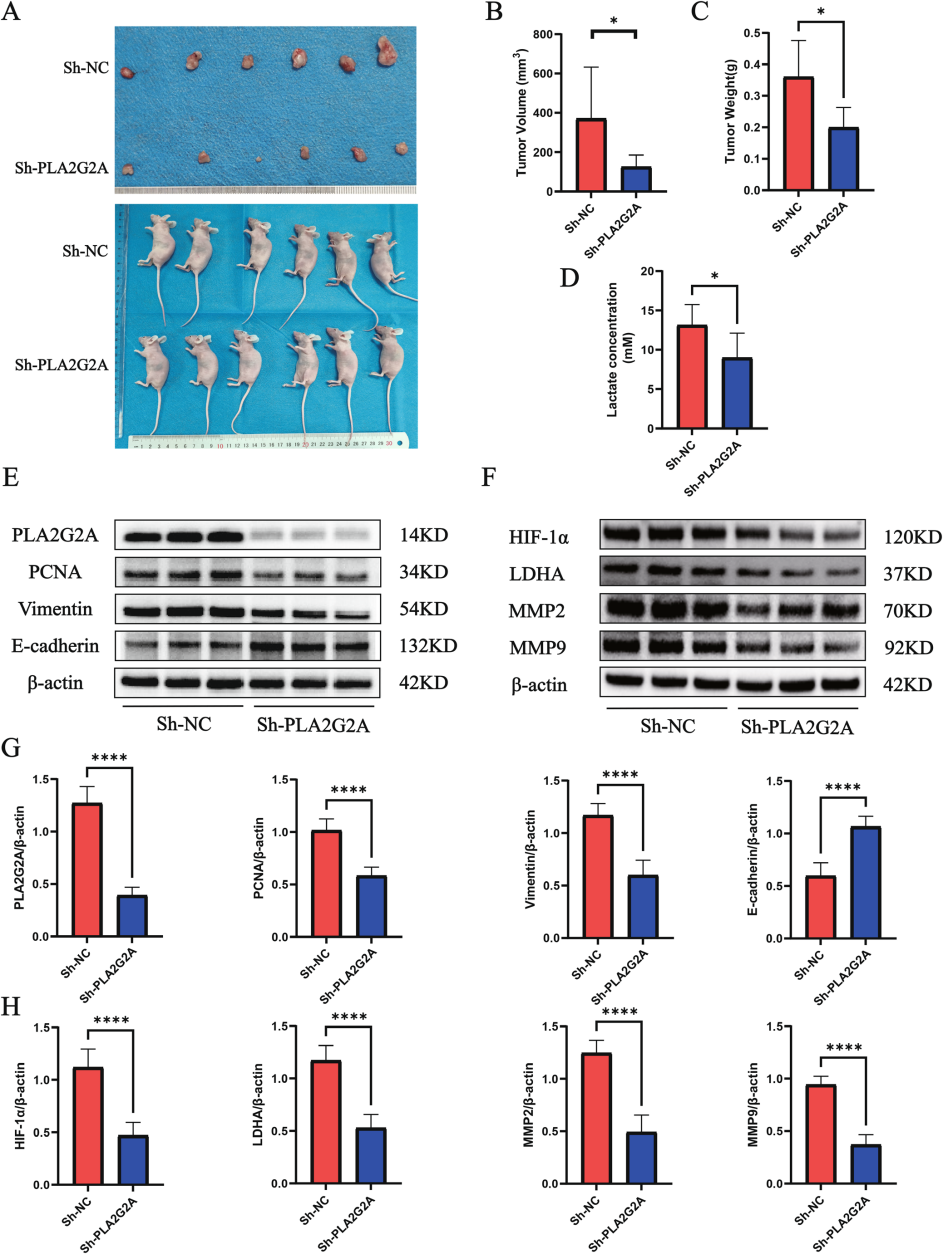
Downregulation of PLA2G2A decreases tumor lactate levels, inhibits tumor growth, and reverses EMT phenotype. (A) Subcutaneous tumorigenesis experiment in mice showing differences in tumor morphology between the Sh‐NC and Sh‐PLA2G2A groups. (B, C) Comparison of tumor volume and weight, showing significantly reduced tumor growth in the Sh‐PLA2G2A group compared to the control group (*p < 0.05). (D) Lactate levels in the Sh‐NC and Sh‐PLA2G2A groups (*p < 0.05). (E, F) Western blot analysis of protein expression in the Sh‐NC and Sh‐PLA2G2A groups. Compared to the control group, PLA2G2A and the proliferation marker PCNA were significantly downregulated, the mesenchymal marker Vimentin was decreased, and the epithelial marker E‐cadherin was upregulated. HIF‐1α, LDHA, MMP2, and MMP9 were also significantly reduced. (G, H) Protein quantification analysis further confirmed the differences, with statistical significance (****p < 0.0001).

Earlier research has investigated the involvement of the genes *VAV3* and *TNFRSF12A* in gliomas [24, 25], while research on *PLA2G2A* in gliomas is limited. Therefore, we focused on studying PLA2G2A. Protein expression analysis across different cell lines showed that PLA2G2A was prominently expressed in several glioma cell lines (Figure 10F,G). To examine PLA2G2A's role in tumor cells, we silenced its expression via shRNA and confirmed knockdown efficiency in U251 and LN229 cells through Western blot (Figure 10H–K). Functional assays demonstrated that reducing PLA2G2A expression notably suppressed the proliferation of U251 and LN229 tumor cells (Figure 10L,M). Immunofluorescence indicated successful construction of stable U251 and LN229 transfected cells (Figure 10N). Additionally, Transwell assays showed that PLA2G2A knockdown significantly reduced U251 and LN229 cell migration and invasion (Figure 10O). Quantitative analysis further showed that, in comparison to the control group, the number of migrating and invading cells was markedly lower in the sh‐PLA2G2A treated group (Figure 10P). To validate the in vitro observations, we delved into the impact of PLA2G2A within a murine subcutaneous tumor experimental setup. We performed tumorigenesis experiments using U251 cells in mice. The findings showed that tumors from PLA2G2A‐silenced cells had significantly lower volume and weight than controls (Figure 11A–C). To investigate the effect of PLA2G2A on lactate metabolism in gliomas, PLA2G2A was knocked down in vitro, and lactate levels were measured. The results showed that lactate concentrations in the supernatants of PLA2G2A knockdown glioma cells were significantly reduced (Figure 11D), further supporting the potential regulatory role of PLA2G2A in lactate metabolism. Western blot results further showed that PLA2G2A knockdown was accompanied by downregulation of the proliferation marker Proliferating Cell Nuclear Antigen (PCNA) and the mesenchymal marker Vimentin, At the same time, the epithelial marker E‐cadherin was increased, indicating a reversal of the epithelial‐mesenchymal transition (EMT) process (Figure 11E,G). After PLA2G2A knockdown, the expression of HIF‐1α was significantly decreased in the knockdown group, further supporting the role of PLA2G2A in lactate‐driven hypoxic responses. The expression of LDHA was also reduced in the PLA2G2A knockdown group, suggesting its regulatory role in lactate metabolism. Additionally, the expression levels of MMP2 and MMP9 were significantly decreased, indicating that PLA2G2A may influence tumor cell invasiveness by regulating extracellular matrix remodeling pathways (Figure 11F,H).

## 4 Discussion

LGG is a diffuse tumor that originates from glial cells within the central nervous system [26]. Although the growth rate of LGG is relatively slow, it often undergoes malignant transformation as the disease progresses, eventually evolving into high‐grade glioma or even glioblastoma (GBM), leading to a poor prognosis [27]. At present, treatment mainly emphasizes maximal tumor resection, followed by chemotherapy, such as temozolomide (TMZ) and radiotherapy. However, due to the recurrence and drug resistance of LGG, the clinical treatment outcomes remain limited [4]. Therefore, systematically identifying novel molecular features and constructing a robust prognostic model is of great significance for improving risk stratification and personalized treatment in LGG.

Lactate, as a typical product of the “Warburg effect,” has been demonstrated to be crucial in the onset and progression of several types of cancer [28]. Tumor cells generate significant amounts of lactate by increasing glycolytic activity, even in the presence of oxygen (aerobic glycolysis). This process not only supplies energy and metabolic intermediates to tumor cells but also results in acidification, immune suppression, and angiogenesis due to the buildup of lactate in the tumor microenvironment (TME) [29]. Research has demonstrated that lactate can suppress T cell and NK cell activity, while promoting the growth of regulatory T cells and myeloid‐derived suppressor cells, facilitating tumor immune evasion [30, 31]. Beyond serving as a metabolic byproduct, lactate acts as the key signaling molecule that modulates critical transcription factors and cellular pathways, including HIF‐1α and NF‐κB [32], ultimately shaping tumor behavior by regulating cancer cell growth, motility, and metastatic potential [33, 34].

In this context, we focused on lactate metabolism–related pathways and systematically analyzed the expression characteristics and clinical significance of lactate metabolism–immune regulation–related genes (LMIRGs) in LGG. Our results show that LMIRGs can effectively stratify patients, with significant differences in survival prognosis, immune cell infiltration, and molecular pathway activation among different subtypes. Through a multi‐step screening process, we ultimately constructed a three‐gene risk signature consisting of *VAV3, TNFRSF12A,* and *PLA2G2A*. This model demonstrated stable and reliable predictive performance in both the TCGA and CGGA cohorts, and its clinical applicability was further enhanced through a nomogram.

**FIGURE 12:**
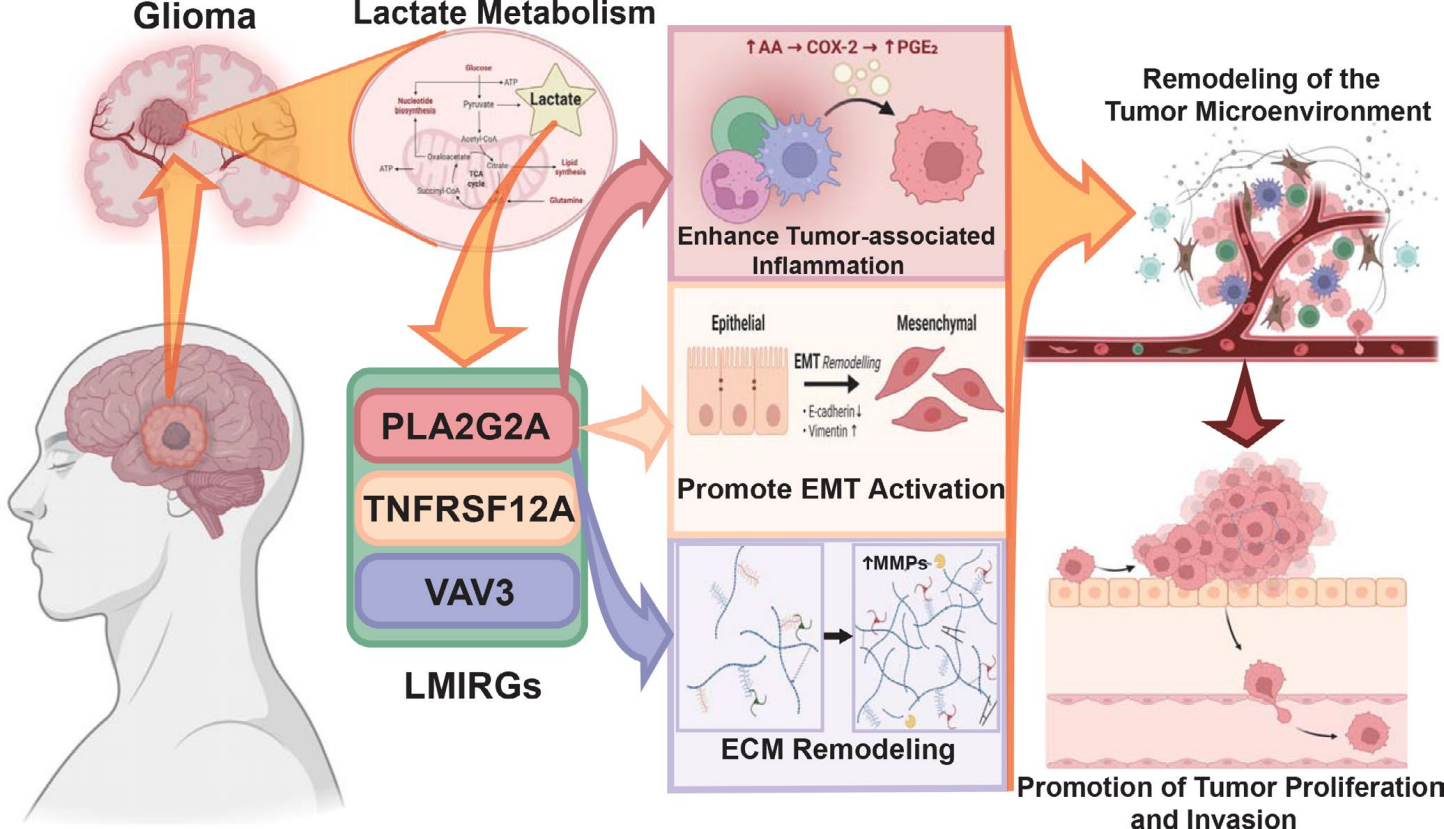
Schematic of lactate metabolism‐immune regulation‐related genes altering the tumor microenvironment in glioma. In glioma, clustering analysis based on lactate metabolism gene sets identified *PLA2G2A, TNFRSF12A,* and *VAV3* as differentially expressed genes included in the predictive model. *PLA2G2A* alters the tumor microenvironment by remodeling the ECM, promoting tumor‐associated inflammation, and activating epithelial‐to‐mesenchymal transition (EMT), thereby enhancing tumor cell proliferation and invasion, ultimately leading to poor prognosis.

It is worth noting that, although the three‐gene signature is not composed of classical lactate metabolism key enzymes, functional enrichment analysis revealed that these genes are primarily involved in immune regulation, extracellular matrix (ECM) remodeling, and inflammatory response, rather than purely metabolic processes. This finding suggests that the tumor‐promoting effects of lactate metabolism in LGG may not solely rely on core metabolic pathways, but rather on the activation of downstream immune modulation and microenvironment remodeling networks, which indirectly drive tumor progression (Figure 12). Therefore, we believe that although this three‐gene model extends beyond the narrow definition of “lactate metabolism genes,” it more accurately captures the critical biological process of lactate metabolism–immune microenvironment interaction, thus holding significant prognostic value.

The interaction between metabolic reprogramming and the immune microenvironment is considered one of the core mechanisms driving tumor malignancy [35], with lactate metabolism serving as a key hub in this process [36]. Consistent with findings from other tumor studies, our results further emphasize the central role of metabolic‐immune interactions in tumor biology [37]. Immunological profiling indicated that individuals in the high‐risk category exhibited significant impairments in immune cell recruitment, particularly marked by depleted levels of CD8+ T cells and dendritic cells. These findings imply that high‐risk patients likely present with immunologically “cold tumor”, potentially compromising their response to immunotherapeutic interventions. Moreover, the at‐risk population exhibited elevated TMB levels and a favorable association with the risk assessment index. Previous studies suggest that although high TMB may increase the production of neoantigens, it is still difficult to translate into clinical benefit in the absence of an effective immune response [38]. Our results further support this notion, suggesting that the prognosis of LGG should be evaluated by considering metabolic status, immune microenvironment, and genetic mutation characteristics in an integrated manner. By integrating TMB with metrics of immune infiltration (such as the presence of CD8+ T cells, Treg cell activity, and M1/M2 macrophage polarization) and immune effector activity (such as cytokine profiles and immune checkpoint activity), a more accurate prognosis can be achieved. Combining TMB with these immune microenvironment markers provides a more comprehensive view of tumor biology and immune response, which can help guide therapeutic decisions, particularly in the context of immunotherapy [39]. Previous studies have shown that the combination of TMB with immune‐related biomarkers significantly improves prognostic accuracy and therapeutic decision‐making in cancers [38, 40]. We propose that similar integrated approaches could be applied to LGG to enable better patient stratification and personalized treatment strategies. We observed notable variations in the responsiveness to chemotherapy and targeted therapies across the different risk groups. Patients in the low‐risk category responded more favorably to fluoropyrimidine and hormone‐targeted drugs, while those in the high‐risk group saw better outcomes with Temozolomide, tyrosine kinase inhibitors (TKIs), and platinum‐based chemotherapy. This indicates that the risk model not only forecasts prognosis but also offers insights for selecting appropriate drugs. Particularly in the realm of precision medicine, this model is anticipated to assist clinicians in developing personalized treatment strategies, enhancing treatment effectiveness while minimizing unnecessary drug exposure.

*PLA2G2A* is implicated in inflammation, lipid homeostasis, and cellular infiltration across several types of tumors [41, 42, 43]. This study validated the tumorigenic role of PLA2G2A in LGG through both in vitro and in vivo experiments. Knockdown of PLA2G2A significantly inhibited cell proliferation, migration, and invasion, and reversed the epithelial‐mesenchymal transition (EMT) process, while also significantly suppressing tumor growth in vivo. After PLA2G2A knockdown, lactate concentrations in the supernatants of glioma cells were significantly reduced, further supporting the potential regulatory role of PLA2G2A in lactate metabolism. Additionally, Western blot analysis showed a significant decrease in the expression levels of MMP2 and MMP9 following PLA2G2A knockdown, suggesting that PLA2G2A may regulate tumor cell invasiveness through modulation of extracellular matrix (ECM) remodeling pathways. Lactate accumulation in the tumor microenvironment typically promotes tumor progression by inducing inflammation and modulating immune cell function. PLA2G2A may influence these processes, indirectly regulating the immune microenvironment and tumor cell migration. Furthermore, the expression of HIF‐1α was significantly decreased in the PLA2G2A knockdown group, further supporting the role of PLA2G2A in lactate‐driven hypoxic responses and metabolic reprogramming [44]. These results suggest that PLA2G2A may participate in lactate‐driven tumor microenvironment remodeling by regulating inflammation and ECM remodeling, thereby promoting the progression of LGG.

In addition to *PLA2G2A*, the other two genes, *VAV3* and *TNFRSF12A*, in our model have also been reported to play important roles in various tumors. *VAV3* belongs to the guanine nucleotide exchange factor family and can regulate the activation of Rho GTPases, thereby affecting cytoskeletal remodeling, adhesion, and migration [45]. Findings indicate a high expression of VAV3 in breast, gastric, and colorectal cancers, which can trigger the PI3K/AKT or MAPK signaling pathways [46, 47], and is associated with tumor cell invasiveness and drug resistance [48, 49, 50]. In gliomas, VAV3 may be involved in tumor cell proliferation, migration, invasion, and the self‐renewal of cancer stem‐like cells [24]. From the perspective of “lactate metabolism–immune microenvironment interaction,” lactate accumulation in the tumor microenvironment (TME) can alter tumor cell motility and immune cell migration/infiltration efficiency through acidification and signal transduction [51]. VAV3, as a core regulator of cytoskeletal dynamics and cell migration [52], may play a downstream effector role in the process of “metabolic stress—enhanced invasiveness—immune evasion,” thus functioning in a coupled manner with lactate‐driven microenvironment remodeling. Moreover, pathways such as PI3K/AKT and MAPK are widely involved in tumor metabolic reprogramming [53], including enhanced glycolysis and the maintenance of immune suppressive phenotypes. This suggests that VAV3 may serve as a critical node linking metabolic state alterations with the transition of invasion/immune phenotypes. *TNFRSF12A* (Fn14) belongs to the TNF receptor superfamily and plays a key role in inflammatory responses and tissue regeneration [54]. Multiple studies have shown that *TNFRSF12A* is overexpressed in gliomas and is closely associated with angiogenesis, invasion, and poor prognosis [25, 55]. Mechanistically, the Fn14 axis can drive the amplification of inflammatory signals, cell survival, and the activation of matrix‐related transcription programs, thereby promoting tumor invasiveness and ECM remodeling [56, 57]. Considering that lactate is not only a metabolic byproduct but also a signaling molecule that activates transcriptional networks such as HIF‐1α and NF‐κB, driving immune suppression and matrix remodeling [58, 59], the inclusion of TNFRSF12A in this study is biologically justified. It may represent a key downstream effector molecule of inflammatory signaling and ECM remodeling programs under lactate‐enriched conditions. Therefore, although *VAV3* and *TNFRSF12A* are not traditional lactate metabolism key enzymes, existing studies suggest that they can influence invasion, migration, inflammatory signaling, and ECM remodeling processes, thus forming a “functionally coupled” relationship with lactate‐driven immune suppressive TME, supporting their inclusion as part of the lactate metabolism–immune regulation‐related gene (LMIRG) signature.

The research does present some drawbacks. For one, the examination was heavily based on the TCGA and CGGA public databases, which could introduce sampling errors. Thus, multicenter prospective studies are needed for further validation. Second, the experimental validation focused only on *PLA2G2A*, while the mechanisms of other genes such as *VAV3* and *TNFRSF12A* have not yet been thoroughly investigated. In addition, the analysis of the immune microenvironment was mainly based on bioinformatics algorithms [60]; future studies could incorporate single‐cell sequencing and spatial transcriptomics for more precise validation. Finally, predictions of drug sensitivity need to be confirmed through clinical trials.

## 5 Conclusion

This research systematically exposes the categorization and predictive significance of LMIRGs in LGG and establishes a reliable three‐gene risk profile. This framework not only accurately forecasts patient outcomes but also captures variations in the immune landscape and therapeutic response, offering crucial insights for clinical risk assessment and tailored treatment strategies. The functional validation of *PLA2G2A* and the supporting literature on *VAV3* and *TNFRSF12A* further emphasize the biological rationale and clinical potential of this model. In the future, integrating multi‐omics data and clinical trials will help facilitate the clinical translation of the LMIRGs risk model and open new avenues for precision treatment of LGG.

## Author Contributions

Xiang Gao and Yi Huang contributed to the conception and design of the study. Enhao Zhang, Liangzhe Wei, He Ren, Meng Sun, Hongqiao Yang, Jianfei Zhang performed experiments and organized the database. Enhao Zhang wrote the first draft of the article. Xiang Gao and Yi Huang reviewed and edited. All authors read and approved the final manuscript.

## Funding

Our study was supported by Ningbo Top Medical and Health Research Program (2022020304), Ningbo Natural Science Foundation (2023J135), and Ningbo Municipal Youth Science and Technology Innovation Leading Talent Project (2025QL015).

## Ethics Statement

The experiments in this study were approved by the Ethics Committee of The First Affiliated Hospital of Ningbo University (No. 2023127A).

## Consent

The authors have nothing to report.

## Conflicts of Interest

The authors declare no conflicts of interest.

## Supporting information


**Dataset S1.**Supporting information.

## Data Availability

The data and materials in this current study are available from the corresponding author on reasonable request.
